# Records for ticks (Acari: Ixodidae) on free-ranging *Coendou spinosus* from State of São Paulo, Brazil

**DOI:** 10.1007/s10493-026-01122-1

**Published:** 2026-03-13

**Authors:** Ticiana Zwarg, Marcelo Bahia Labruna, Mariana Morgado Hereny, Thaís Caroline Sanches, Mayra Hespanol Frediani, Vanessa Caldeira Olivares, Melissa Prosperi Prosperi, Felipe Almeida Lucato, Giovanna Silva Alves de Lima, Adriana Marques Joppert, Sergio Mello Novita Teixeira, Alex Junior Souza de Souza, Sandro Marques, Jean Carlos Ramos Silva, Barbara Antonieta Ribeiro Pilão, Erika Sayuri Kaihara, Lilian Rose Marques de Sá

**Affiliations:** 1 School of Veterinary Medicine and Animal Science, University of São Paulo, São Paulo, Brazil; 2 Wildlife Management and Conservation Center, Wildlife Division, Green and Environment Secretariat, São Paulo City Hall, Brazil; 3 Postgraduate Program in One Health, Santo Amaro University, São Paulo, Brazil; 4 Laboratory of Identification and Research of Synanthropic Fauna, Division of Zoonosis Surveillance, COVISA, SMS, São Paulo City Hall, Brazil; 5 Associação Mata Ciliar, Jundiaí, SP Brazil; 6 Núcleo da Floresta - Wildlife Rehabilitation Center, São Roque, SP Brazil; 7 Wildlife Rehabilitation Center of Barueri, Barueri City Hall, Barueri, SP Brazil; 8 Departamento de Patologia, Faculdade de Medicina Veterinária e Zootecnia, Universidade de São Paulo, Av. Prof. Dr. Orlando Marques de Paiva, 87, Cidade Universitária, São Paulo, CEP 05508-270 SP Brasil

**Keywords:** Amblyomma, Erethizontidae, Parasitology, Rodents, Hard ticks

## Abstract

Ticks and tick-borne diseases have spread since the mid-twentieth century largely due to major anthropogenic changes impacting natural ecosystems. The *Coendou spinosus* is a medium sized rodent found in the southeast of Brazil. There is little information about the presence and richness of ticks on porcupines. The objective of this study was to identified ticks collected from *C. spinosus* received at a triage center of wildlife in the city of São Paulo, from 1996 to 2025 (almost 30 years). Ticks from a total of 100 free-ranging porcupines were collected. A total of 223 ticks in various life stages were analyzed. Fifty-eight larvae, 17 nymphs, and 148 adults were identified. Of these, 89 were males and 59 were females. Some animals presented mixed infestations, with more than one tick species. The most prevalent species was *Amblyomma longirostre*, with samples from 86 animals, followed by *A. parkeri* (18 animals), *A. dubitatum* (1 animal), *A. ovale* (1 animal), *A. sculptum* (1 animal), and *Haemaphysalis juxtakochi* (1 animal). To the best of our knowledge, this is the first report of the presence of *Haemaphysalis juxtakochi* parasitizing this porcupine species. Additionally, we highlight the observation of a male of *A. longirostre* attached to the porcupine’s spine. Ticks are important vectors of several pathogens causing zoonotic diseases. This work contains information that can contribute to knowledge and conservation of porcupines, and to the development of environmental surveillance strategies.

**Supplementary Information:**

The online version contains supplementary material available at 10.1007/s10493-026-01122-1.

## Introduction

Ticks and tick-borne diseases have spread since the mid-twentieth century largely due to major anthropogenic changes impacting natural ecosystems (Fish 2022). Studies provide evidence that climate change has contributed to the expanded range of ticks (Beard et al. 2016). Mammals are more parasitized by ticks than birds, reptiles, and amphibians. Some species feed only on a narrow range of host groups; others are host species specific, and others are less selective, feeding a wide range of animals (Sonenshine 1991). Among mammals, rodents are preferred for the immature stages of ixodid ticks (Barros-Battesti et al. 2024).

Neotropical porcupines are natural hosts of lice (*Eutrichophilus* sp.) (Brum et al. 2003; Lignon et al. 2023), mites (Busi et al. 2022) and are also host for ticks (Labruna et al. 2009; Dantas-Torres 2010). *Amblyomma* spp. parasitizes a wide variety of domestic and wild animals, and approximately 100 species are found predominantly in tropical and subtropical areas (Zajac and Conboy 2012). In Brazil, *Coendou spinosus* has been reported as hosts for *Amblyomma longirostre* (Barros and Baggio 1992; McIntosh et al. 2015; Valente et al. 2022; Acosta et al. 2024; Martins et al. 2025), A. *parkeri* (Labruna et al. 2009; Martins et al. 2013, 2017; González et al. 2017; Valente et al. 2022; Luz et al. 2023), *A. ovale* (Arzua et al. 2005), A. *sculptum* (Arzua et al. 2005) A. *dubitatum* (Acosta et al. 2024) and *Rhipicephalus microplus* (Valente et al. 2022). Porcupines maintain a unique ecological relationship with ticks, particularly with *A. longirostre* and *A. parkeri*, which are frequently associated with Erethizontidae hosts (Labruna et al. 2009; Luz et al. 2018).

The close ecological association between porcupines and human settlements raises significant concerns about potential zoonotic spillover pathways (Friant et al. 2025; Martins et al. 2025). In the Neotropical region, hard ticks are the main arachnid vectors of pathogens for humans, domestic and wild animals (Martins et al. 2024). Therefore, some species are relevant to animal health and public health (Guglielmone and Robbins 2018; Nogueira et al. 2022), since they transmit bacteria such as *Anaplasma*, *Borrelia*, *Ehrlichia*, and *Rickettsia*, as well as protozoa such as *Babesia*,* Cytauxzoon*,* Hepatozoon*,* Rangelia* and *Theileria* (Barros-Battesti et al. 2006; Nava et al. 2017). For porcupines, *Eutrichophilus lice*,* A. sculptum*, and particularly *A. longirostre* ticks may play a role in Brazilian porcupinepox virus (BPoPV) transmission (Martins et al. 2025).

The frequency of rodent-borne diseases has been steadily rising in recent years due to several drivers, including the emergence of new pathogens, environmental shifts, climatic changes, and anthropogenic activities, such as urbanization, deforestation, and agricultural intensification (Shehata et al. 2025). Threat reduction, for both endemic and emerging rodent-borne diseases, requires understanding the ecological mechanisms driving spillover and applying these insights for prevention (Friant et al. 2025). In this context, it includes knowledge about rodent ticks, which are involved in the indirect transmission of diseases. Besides, rodents are poorly represented in the collections of Brazilian zoos, and they have been little considered in medical and zootechnical research (Lange and Schimidt 2014). In our knowledge, this is the first study focused on ticks from wild neotropical porcupine, *C. spinosus*. The objective of this study is to present the identified ticks collected from *C. spinosus* received at a triage center of wildlife in the city of São Paulo, from 1996 to 2025.

## Materials and methods

This study has authorization for data collection and laboratory analysis of biological materials from the following institutions: SISBIO (nº 79891-2); Ethics Committee on the Use of Animals of the School of Veterinary Medicine and Animal Science of the University of São Paulo (CEUA/FMVZ nº 1221260122-ID 009657); and Technical Committee for Scientific Evaluation of the Secretariat for Green and Environment of the Municipality of São Paulo (nº 6027.2021/0012190-2). The activity of access to Genetic Heritage was registered in SisGen, in compliance with the provisions of Law No. 13,123/2015 and its regulations (registration number: A429CFC).

The current report comprises identification records of larvae, nymphs and adult ticks that were collected from *C. spinosus* in the Southeast region of Brazil. The Wildlife Management and Conservation Center, Wildlife Division, Green and Environment Secretariat, São Paulo City Hall (DFS, −23.421479,−46.787048) is a triage center of wildlife in São Paulo, Brazil, and develops actions to protect and conserve the wildlife of the municipality, metropolitan region, and that originating from seizures, in actions to combat trafficking. One of its main activities involves veterinary care with laboratory support aimed at the recovery of animals rescued and sent to the service. Animals brought to the service are generally found injured or in conflict situations and are taken by residents or police officers.

From July 1996 to June 2025 (29 years), DFS received 381 *C. spinosus*. Ticks from a total of 100 individual hosts were collected, which represents a sampling effort of 26.25% of the total number of animals received in the period. Unfortunately, the remaining 281 animals were not investigated for the presence of ticks due to the large number of animals received and few professionals working there.

Tick ​​collections were performed during the initial examination of the animal, as soon as it was received for care, or during the necroscopic examination, when dead. All specimens were manually collected from different *C. spinosus* and preserved in 70% ethanol until morphological analysis. The anatomical locations where ticks were found attached to porcupines were not recorded in a systematic way in all situations, but it was done for 100 porcupines. Samples were morphologically analyzed and identified based on pictorial and dichotomic taxonomic keys for the identification of the genus, the species and different life stages of the ticks (Barros-Battesti et al. 2005, 2006; Labruna et al. 2009; Martins et al. 2010, 2013).

Identifications were carried out at the Laboratory of Identification and Research of Synanthropic Fauna of the Center for Zoonosis Control – São Paulo City Hall (LABFAUNA-DVZ-PMSP) and in the Laboratory of Parasitic Diseases of the School of Veterinary Medicine and Animal Science of University of São Paulo.

Porcupines were analyzed for date of entry into the service, origin, age, and sex. The age of the animals was estimated according to the morphological characteristics based on the eruption of the upper incisor teeth and the maturation of the coat, according to Voss and Angermann (1997) and Caldara-Junior and Leite (2012). Sexing was performed by exposing genitals. The mean intensity of ticks on porcupines was determined for each tick species according to the method of Bush and coworkers (1997). In this case, mean intensity was calculated by dividing the total number of ticks by the number of infested hosts (*n* = 100).

The origin of the tick-infested porcupines were analyzed according to the 11 geopolitical regions of the state: São Paulo (SP), Sorocaba (SO), Bauru (BA), Marília (MA), Presidente Prudente (PP), Araçatuba (AR), São José do Rio Preto (SJRP), Ribeirão Preto (RB), Araraquara (ARR), Campinas (CP), and São José dos Campos (SJC).‌

## Results

A total of 223 specimens of ticks, comprising 58 larvae, 17 nymphs and 148 adults (89 males and 59 females) were collected from the 100 porcupines. Some animals presented mixed infestations, with two different tick species (10 porcupines) or even three tick species at the same time (one animal). The most frequent and abundant tick species was *Amblyomma longirostre*, with samples from 86 animals (86/100; 86%), followed by *A. parkeri* (18/100; 18%), *A. dubitatum* (1/100; 1%), *Amblyomma ovale* (1/100; 1%), *Amblyomma sculptum (*1/100; 1%) and *Haemaphysalis juxtakochi* (1/100; 1%). From 4 animals (4/100; 4%), the *Amblyomma* species was not identified (*Amblyomma* spp.). Table 1 shows the distribution of ticks on porcupines.

**Table 1:** Infestation levels caused by tick species on 100 individual specimens of porcupines (*Coendou spinosus*) from the São Paulo Metropolitan region and surrounding cities in Brazil, from July 1996 to June 2025

Tick species	Number of ticks according to stage						No. infested porcupines *n* = 100 (%)	Mean intensity
	Larvae	Nymphs	Males	Females		Total		
*Amblyomma longirostre*	35	8	72	58	173		86 (86)	2.0
*Amblyomma parkeri*		4	17	1	22		18 (18)	1.2
*Amblyomma sculptum*		2			2		1 (1)	2.0
*Amblyomma dubitatum*		1			1		1 (1)	1.0
*Amblyomma ovale*		1			1		1 (1)	1.0
*Haemaphysalis juxtakochi*		1			1		1 (1)	1.0
*Amblyomma* spp.	23				23		4 (4)	5.8
Total	58	17	89	59	223		100 (100)	2.2

Overall, mean intensity was 2.2 ticks/infested porcupine, with *A. longirostre* and *A. parkeri* presenting the highest mean intensity values, 2.0 ticks/infested host (the mean intensity value of 5.8 for *Amblyomma* spp. larvae is not considered in this comparison because it could represent two or more unidentified *Amblyomma* species. (Table 1). Detailed information (geographical location, date of collection, age of host, number of ticks per stage and species) for each of the tick-infested porcupine is given in Supplemental Table S1.

**Fig. 1:**
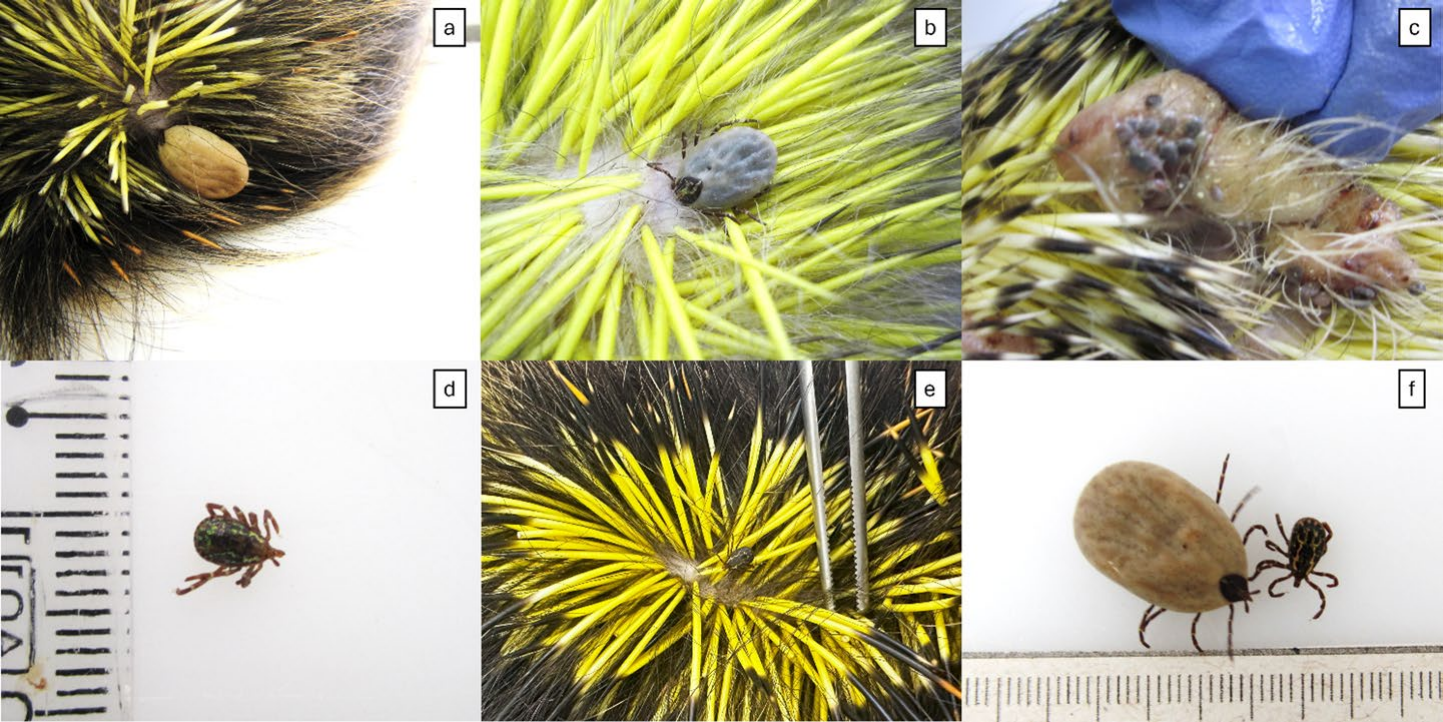
Ticks found on *Coendou spinosus*. (a) Female of *Amblyomma longirostre*, in dorsal region (ID 115754); (b) Female of *Amblyomma longirostre*, dorsal region (ID 101799); (c) Larvae of *Amblyomma longirostre*, ear (ID 99521); (d) Male of *Amblyomma parkeri* (ID 106400), in the same of animal of item a; (e) Male of *Amblyomma longirostre*, dorsal region (ID 126353); (f) Female (left) and male (right) of *Amblyomma longirostre* (ID 106900)

**Fig. 2:**
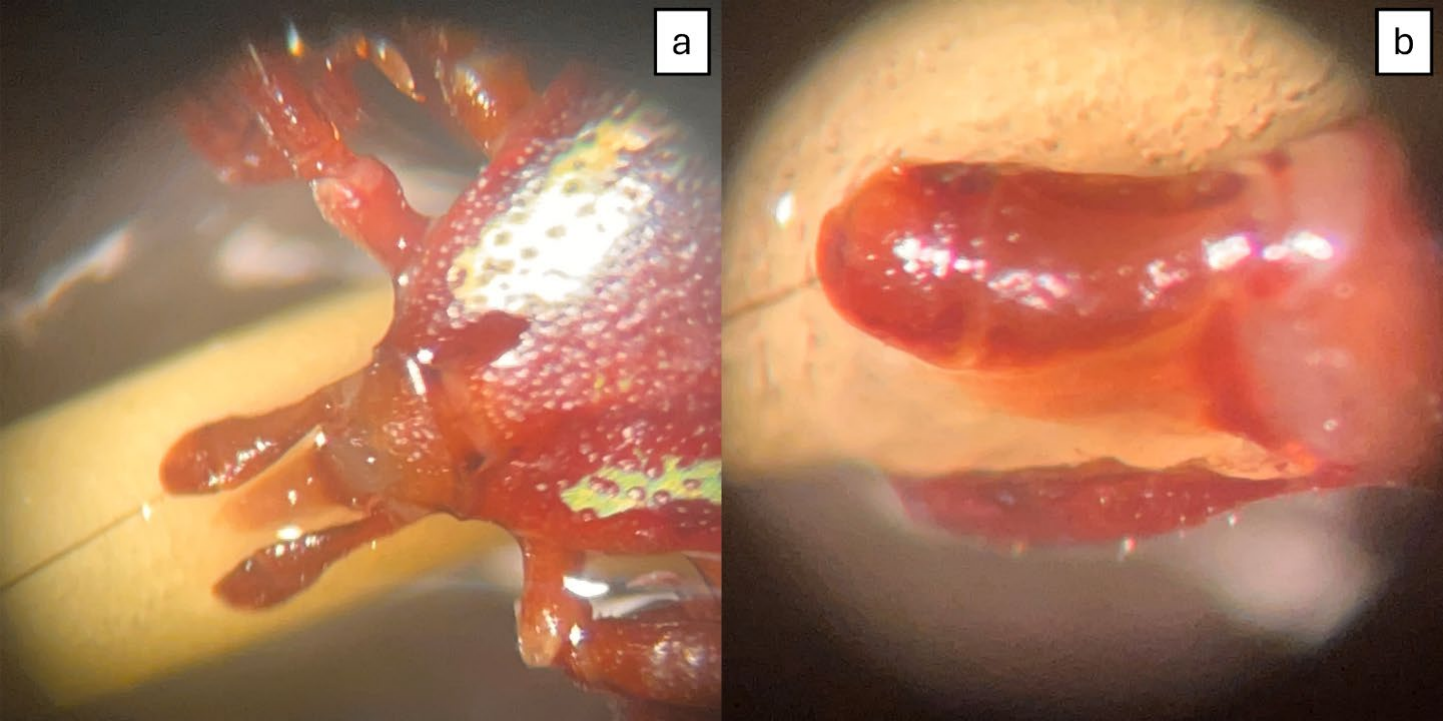
*Amblyomma longirostre* male attached to the porcupine’ spine (ID 138561). (a) Tick legs without contact with the host’s skin; (b) Detail of tick capitulum attached to the porcupine spine

**Fig. 3:**
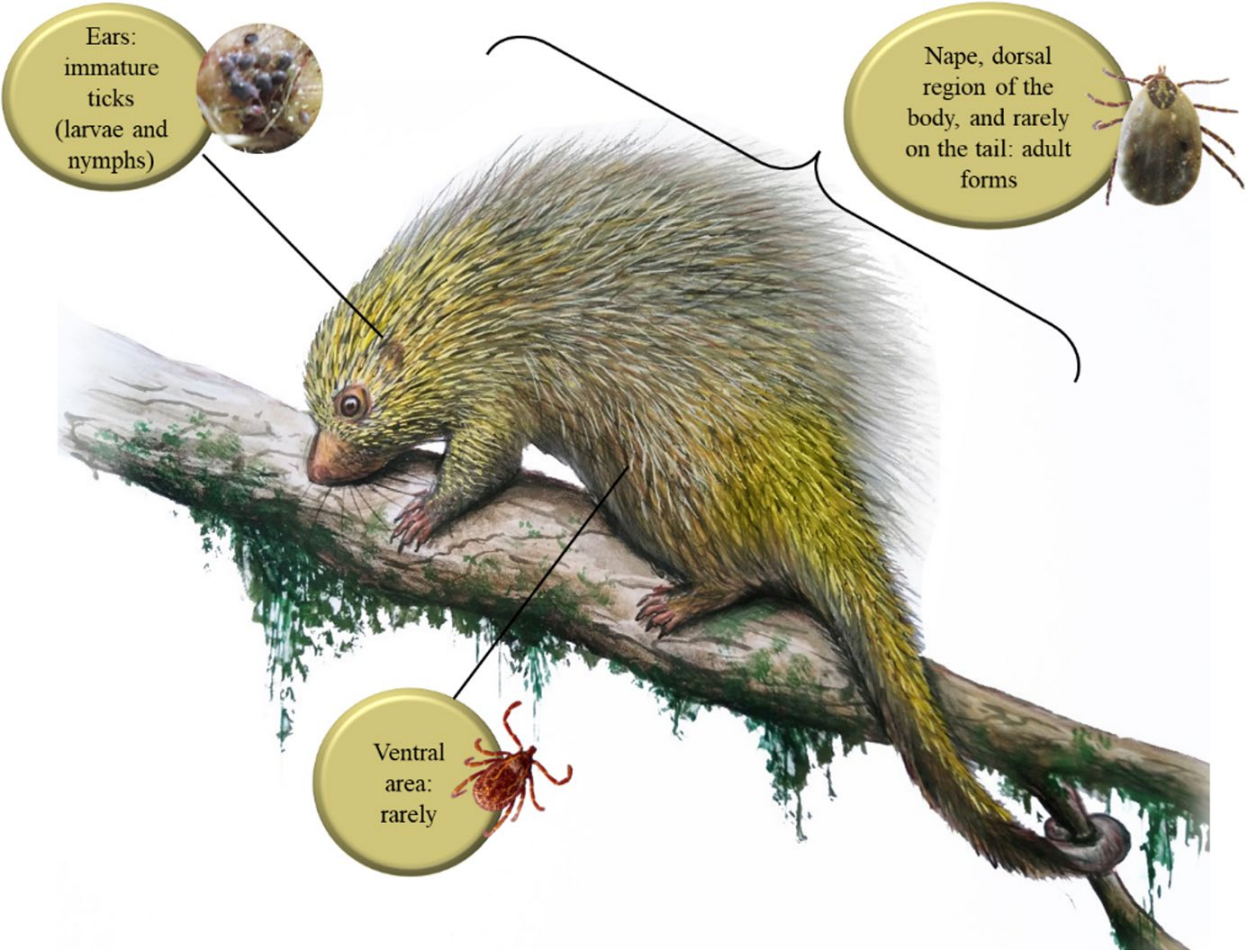
Preferred areas of the body of porcupine for infestation of ticks. Observation of tick infestation at various life stages on the body of *C. spinosus*. Illustrator: Fernando Igor

Adult ticks were mostly found on the nape, dorsal region of the body, and rarely on the tail (Fig. 1 – a, b, e). The immature ticks (larvae and nymphs) preferred to settle on the ears (Fig. 1 – c). No ticks were found in the ventral, cervical, and genital regions. One adult tick was found on the ventral surface of the thoracic member. One of the males of *A. longirostre* was attached to the porcupine’s spine (Fig. 2), and the tick legs were free, with no direct contact with host skin. Figure 3 illustrates these preferred areas of the body of porcupine for infestation of ticks and observations about tick infestation at various life stages on the body of *C. spinosus*. Regarding the host profile, ticks were collected from 87 adult porcupines (87/100 − 87%), 7 juveniles (7/100–7%), and 5 pups (5/100–5%). For one animal, age information was not available (1/100–1%). Fifty-two animals were females (52/100 − 52%) and 31 animals were males (31/100 − 31%). The sex of 17 animals was not identified (17/100 − 17%).

**Fig. 4:**
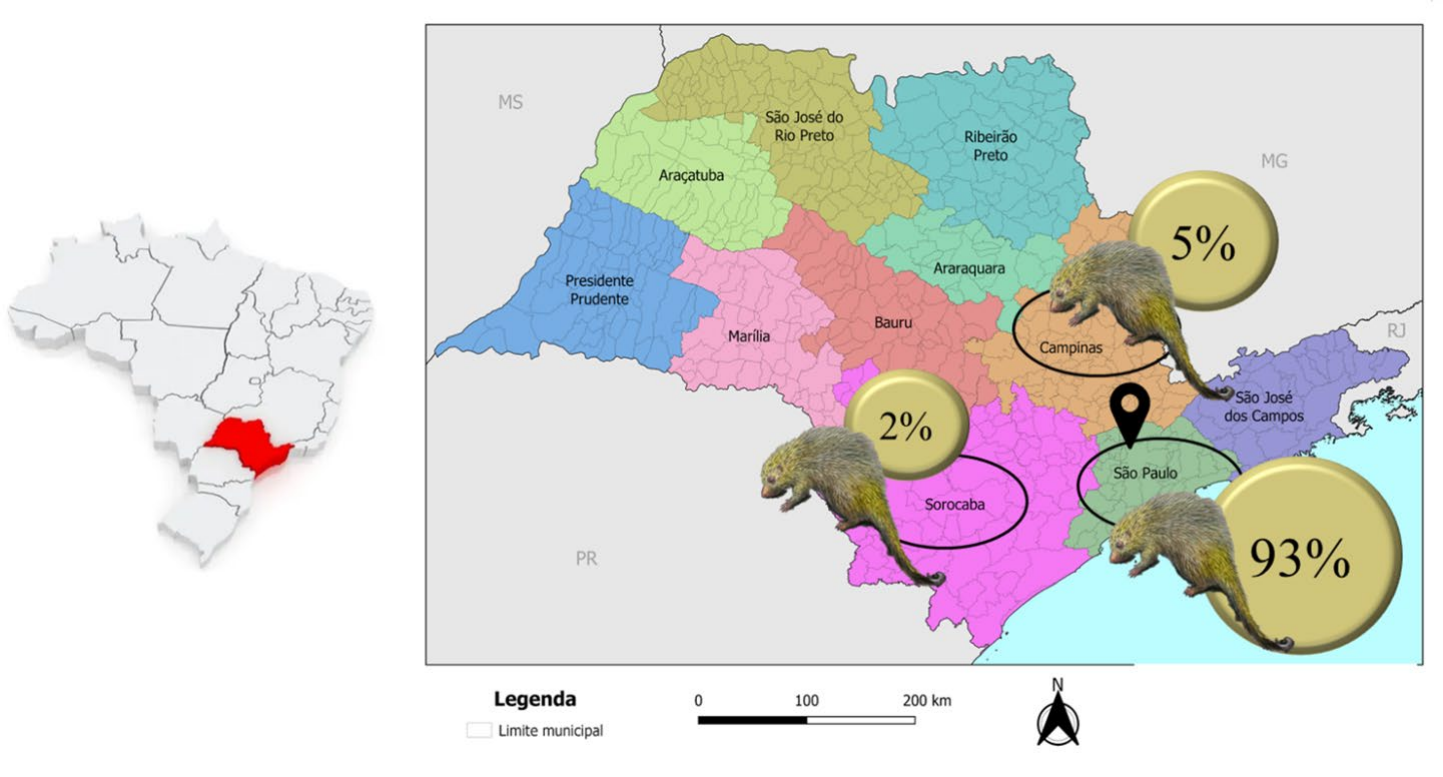
Origin of *Coendou spinosus* analyzed in the study, according to the geographic division into geopolitical regions. The icon  demonstrates the location of the Wildlife Management and Conservation Center, Wildlife Division. Adapted from: https://geoftp.ibge.gov.br/organizacao_do_territorio/divisao_regional/divisao_regional_do_brasil/divisao_regional_do_brasil_em_regioes_geograficas_2017/mapas/35_regioes_geograficas_sao_paulo.pdf

Most of the tick-infested animals came from the city of São Paulo (64/100 − 64%), followed by the cities of Franco da Rocha, with 7 animals (7/100–7%), Cotia (4/100–4%), Caieiras and Jundiaí, with 3 animals each (3/100–3%) and Diadema and Embu das Artes, with 2 animals each (2/100–2%). The cities of Atibaia, Barueri, Cajamar, Carapicuíba, Francisco Morato, Ibiúna, Itapecerica da Serra, Itapevi, Louveira, Mairiporã, Osasco, Santana de Parnaíba, Sorocaba, Taboão da Serra and Vargem Grande Paulista participated with 1 animal each (1/100–1%). All these cities are located close to São Paulo city at a distance ranging from 15 (Osasco) to 100 km (Sorocaba). Considering the classification of geopolitical regions, 93 hosts (93%) came from the São Paulo region, 5 (5%) were collected from the Campinas region and only 2 (2%) from the Sorocaba region (Fig. 4).

Of the animals found in São Paulo city, the corresponding local geopolitical zones were: West Zone, with 27 animals (27/100 − 27%); South Zone, with 12 animals (12/100 − 12%); North Zone, with 13 animals (13/100 − 13%) and East Zone, with 9 animals (9/100–9%). These areas include the parks where the porcupines were found, in the city of São Paulo. Twenty-seven of the 64 animals found in São Paulo came from green areas as parks (27/64–42.18%). The parks with the largest number of revised animals were Anhanguera Municipal Park (located in West Zone of São Paulo − 8 animals − 8/27–29.63%), Juquery State Park (located in the city of Franco da Rocha − 4 animals − 4/27–14.81%) and Alberto Löfgren State Park ((located in North Zone of São Paulo − 3 animals − 3/27–11.11%). There was no information for the origin of 5 animals from São Paulo (5/64–7.81%).

## Discussion

In the present study, *C. spinosus* was found infested by six tick species: *Amblyomma longirostre*,* A. dubitatum*,* A. ovale*,* A. parkeri*,* A. sculptum*, and *Haemaphysalis juxtakochi* within the São Paulo Metropolitan region and surrounding cities. To the authors’ knowledge, we provide the first record of *H. juxtakochi* parasitizing *C. spinosus*. This publication also represents the first observation of ticks on this porcupine species in the metropolitan region of São Paulo.

Among the tick species recorded, A. *longirostre* was the species with the largest number, and it was collected from 86 porcupines. This tick species is widely distributed throughout Brazil, but also in other countries. In South America, *A. longirostre* has been reported from Argentina, Bolivia, Brazil, Colombia, French Guiana, Paraguay, Uruguay, Venezuela (Guglielmone et al. 2003; Venzal et al. 2003) and Peru (Nava et al. 2010). The adult stage usually parasitizes rodents of the family Erethizontidae (porcupines), and the immature stages parasitize birds mostly of the order Passeriformes (Guglielmone et al. 2014); recent surveys by LABFAUNA showed that *A. longirostre* nymphs were collected from 21 different bird species monitored by the Wildlife Division/SVMA (unpublished data). The data is in accordance with the fact that porcupines of the family Erethizontidae, including *C. spinosus*, are indeed the most important hosts for the adult stage of *A. longirostre* (Nava et al. 2010, 2017) and could also play an important role as host for immature forms of *A. longirostre* as pointed before (Luz et al. 2018). In the Amazon state, it has been recorded parasitizing *Coendou* sp., *Coendou prehensilis* and *Coendou nycthemera* (Luz et al. 2018). Few records of this tick species have been made on mammals other than porcupines (Barros et al. 2024); however, there are five records of specimens identified by LABFAUNA collected from humans, with three reports of bites by this tick (adults, one male and two females - unpublished data), it may highlighted potential hidden risk for tick-borne pathogen transmission.

The second most collected tick species from porcupines was *A. parkeri*, with 18 infested hosts. The main hosts for this species are members of the Order Rodentia (Erethizontidae Family) for adults; Rodentia (Erethizontidae) and neotropical Primates (Atelidae) for nymphs; and Passeriformes (several families) for larvae (Barros-Battesti et al. 2024). It is an endemic species of Brazil, with records of human parasitism by nymphs in the Brazilian territory (Nogueira et al. 2022). LABFAUNA has two records of *A. parkeri* nymphs on humans, both with reports of parasitism (unpublished data). In monitoring carried out by the Wildlife Division/SVMA, *A. parkeri* nymphs were collected on birds (*Turdus rufiventris*,* Chaetura meridionalis*,* Megascops choliba*) and mammals (*Alouatta clamitans*,* Didelphis aurita*), identified by LABFAUNA (unpublished data).

*A. dubitatum*,* A. ovale*,* A. sculptum* and *H. juxtakochi* were rarely found in the present study, with only one individual host for each tick species. *A. sculptum* and *A. dubitatum* are common in natural or anthropogenic areas where capybaras (*Hydrochoerus hydrochaeris*) occur (Barros-Battesti et al. 2024). A female adult porcupine from the Jardim Peri neighborhood, near Cantareira State Park in northern São Paulo city, was the host for an *A. dubitatum* nymph. The porcupine in which *A. sculptum* was found was also a female, from Varginha Natural Park, located in the Alto Tietê River Basin, which has many flooded areas. The main hosts for *H. juxtakochi* are artiodactyls (Cervidae) for adults; Passeriformes (several families) and Mammalia (several orders) for immatures in the Neotropical region (Barros-Battesti et al. 2024). It can be found that parasitizing rodents from the families Cricetidae, Dasyproctidae, Erethizontidae and Sciuridae (Nava et al. 2017). The host of this tick was a male adult porcupine, from Embu das Artes city.

Male adults corresponded to the largest number sampled, with 89 individuals, followed by adult females (59), larvae (58) and nymphs (Martins et al. 2017). In the literature, there are a few reports of *A. longirostre* nymphs on porcupines of at least four different species, including *C. spinosus* (Fonseca 1933; Nava et al. 2010; Gonzalez et al. 2017; Teixeira et al. 2017). All together, these results suggest that porcupines might also play an important role as hosts for immature stages of *A. longirostre* (Luz et al. 2018). Indeed, the obvious difficulty of collecting immature ticks (small specimens) on the skin of a porcupine might have contributed to limited number of field records (Luz et al. 2018), which perhaps also explains the low number of nymphs found here.

Our finding of an *A. longirostre* male attached to porcupine spine is preceded by Fonseca (1933), who reported *A. longirostre* males attached to the spine of two *S. villosus* porcupines, and more recently by Luz and coworkers (2018), who reported three *A. longirostre* females attached to *C. subspinosus* spines. Here, we found a male with this behavior. Fonseca (1933) speculated that the tick hypostome would penetrate the spine until deeper enough to feed on liquids inside the spine. However, Luz and coworkers (2018) demonstrated that hypostome did not break the external surface of the spine and suggested that ticks attach to the spine for a short period until they can find a more suitable site for blood feeding. Interestingly, the tick behavior of attaching the spine of porcupines has also been confirmed to occur with *A. parkeri* (Luz et al. 2018).

The origin of the porcupines in this study is closed and related to the location of the Wildlife Triage Center, located in Anhanguera Park, west region of São Paulo. Most of the animals sampled came from this region and its surroundings. The predominant biome in the region encompassing the cities of São Paulo, Campinas, and Sorocaba is the Atlantic Forest. The original vegetation of these areas is mostly composed of Semi-deciduous Seasonal Forest, with some fragments of Cerrado (Brazilian savanna). Due to intense urbanization and historical economic cycles (sugar cane, coffee), most of the original vegetation cover has been suppressed. Currently, the state of São Paulo has about 22.9% of its original cover, and cities like Sorocaba have approximately 16.68% of their natural vegetation remaining (Sanquetta 2008). The population of the State of São Paulo is approximately 44.4 million inhabitants (IBGE 2023); these termites live near the human population in the most urbanized areas. We reported a growing increase in the number of porcupines received in the Wildlife Management and Conservation Center, Wildlife Division, Green and Environment Secretariat, São Paulo City Hall, in the years 2007 to 2022, and the causes include the expansion of deforestation and the fragmentation and reduction of natural habitats by anthropogenic activities (Zwarg et al. 2024). The growing number of cases of poxvirus in *C. spinosus* in this region of the city (Zwarg et al. 2024, 2025) and the recent possible association with ticks as vectors responsible for transmission (Martins et al. 2025) raises an alert for the scientific community working in the conservation of neotropical porcupines.

## Conclusion

The ticks that commonly infest free ranging *Coendou spinosus* residing in the city of São Paulo and surrounding areas belong to the genus *Amblyomma*, the main one being *Amblyomma longisrostre*. We provide the first record of *H. juxtakochi* parasitizing *C. spinosus*. The information obtained in this work can contribute to knowledge and conservation of porcupines, comparative ecology studies of ticks, and to the development of environmental surveillance strategies.

## Supplementary Information

Below is the link to the electronic supplementary material.

Supplementary Material 1

## Data Availability

The datasets generated during and/or analyzed during the current study are available from the corresponding author on reasonable request.

## Declarations

### Footnote

this article originated from a thesis.
