# Supplementary material for: Records for ticks (Acari: Ixodidae) on free-ranging Coendou spinosus from State of São Paulo, Brazil
Source: Exp Appl Acarol. 2026 Mar 13;96(3):33. doi: 10.1007/s10493-026-01122-1 (PMC12987818; doi:10.1007/s10493-026-01122-1)
Supplement: Supplementary file 1 — Supplementary Material 1 [file 10493_2026_1122_MOESM1_ESM.docx]

Supplemental Table S1

List of 100 porcupines (*C. spinosus*) evaluated for the presence of ticks, received at the Wildlife Division (DFS), 1996-2025.

|  | **REGISTER** | **DFS ENTRY** | **AGE** | **SEX** | **PARK** | **ZONE** | **CITY** | **GEOPOLITICAL REGIONS** | **LARVAE** | **NIMPH** | **M** | **F** | ***TICK* SPECIE*S*** |  |
| --- | --- | --- | --- | --- | --- | --- | --- | --- | --- | --- | --- | --- | --- | --- |
|  | 4711 | 22/07/1996 | J | F | TEP | E | São Paulo | SP | 10 | 0 | 0 | 0 | *Amblyomma sp* | |
|  | 7341 | 19/08/1997 | A | F | AP | W | São Paulo | SP | 1 | 0 | 0 | 0 | *Amblyomma sp* | |
|  | 7963 | 13/11/1997 | A | NI | - | - | Caieiras | SP | 10 | 0 | 0 | 0 | *Amblyomma sp* | |
|  | 8168 | 15/12/1997 | A | F | - | N | São Paulo | SP | 0 | 0 | 1 | 1 | *A. longirostre* | |
|  | 31079 | 28/12/2006 | J | NI | - | - | Carapicuíba | SP | 0 | 0 | 1 | 1 | *A. longirostre* | |
|  | 33841 | 05/06/2008 | A | NI | - | - | Itapevi | SP | 0 | 0 | 2 | 1 | *A. longirostre* | |
|  | 36683 | 31/03/2009 | A | NI | ALSP | N | São Paulo | SP | 0 | 0 | 1 | 0 | *A. parkeri* | |
|  |  |  |  |  |  |  |  |  | 0 | 0 | 3 | 0 | *A. longirostre* | |
|  | 41313 | 18/10/2010 | J | M | - | E | São Paulo | SP | 0 | 0 | 0 | 1 | *A. longirostre* | |
|  | 52401 | 13/09/2013 | A | M | - | S | São Paulo | SP | 0 | 0 | 1 | 0 | *A. longirostre* | |
|  | 54061 | 06/01/2014 | A | F | - | S | São Paulo | SP | 0 | 0 | 0 | 2 | *A. longirostre* | |
|  | 54880 | 01/04/2014 | A | M | CP | E | São Paulo | SP | 0 | 0 | 1 | 0 | *A. longirostre* | |
|  | 55955 | 04/09/2014 | A | F | - | E | São Paulo | SP | 0 | 0 | 0 | 1 | *A. longirostre* | |
|  | 56234 | 19/09/2014 | C | NI | - | - | Ibiúna | SO | 0 | 1 | 0 | 0 | *A. longirostre* | |
|  | 56496 | 15/04/2015 | A | F | - | W | São Paulo | SP | 0 | 0 | 0 | 1 | *A. longirostre* | |
|  |  |  |  |  |  |  |  |  | 0 | 1 | 0 | 0 | *A. parkeri* | |
|  | 57611 | 01/12/2014 | J | F | AP | W | São Paulo | SP | 0 | 0 | 1 | 1 | *A. longirostre* | |
|  | 60621 | 14/08/2015 | A | NI | - | N | São Paulo | SP | 0 | 0 | 1 | 0 | *A. parkeri* | |
|  | 60927 | 04/09/2015 | C | F | - | W | São Paulo | SP | 0 | 0 | 0 | 2 | *A. longirostre* | |
|  | 60979 | 11/09/2015 | A | F | - | S | São Paulo | SP | 0 | 0 | 1 | 0 | *A. parkeri* | |
|  |  |  |  |  |  |  |  |  | 0 | 0 | 0 | 1 | *A. longirostre* | |
|  | 62861 | 11/12/2015 | A | F | JSP | W | São Paulo | SP | 0 | 0 | 1 | 0 | *A. parkeri* | |
|  | 64123 | 14/03/2016 | A | F | - | E | São Paulo | SP | 2 | 0 | 0 | 0 | *Amblyomma sp* | |
|  | 64167 | 22/03/2016 | A | F | AP | W | São Paulo | SP | 0 | 0 | 3 | 2 | *A. longirostre* | |
|  | 64947 | 20/07/2016 | A | F | NI | NI | São Paulo | SP | 0 | 1 | 0 | 0 | *A. longirostre* | |
|  | 65086 | 07/08/2016 | A | F | NI | NI | São Paulo | SP | 0 | 0 | 2 | 0 | *A. longirostre* | |
|  | 65852 | 23/09/2016 | A | F | NI | NI | São Paulo | SP | 0 | 0 | 4 | 0 | *A. parkeri* | |
|  | 67102 | 08/11/2016 | A | F | - | - | Diadema | SP | 0 | 0 | 0 | 1 | *A. longirostre* | |
|  | 70540 | 30/06/2017 | A | F | - | W | São Paulo | SP | 0 | 0 | 1 | 0 | *A. parkeri* | |
|  | 70875 | 06/08/2017 | A | NI | - | W | São Paulo | SP | 0 | 0 | 0 | 1 | *A. longirostre* | |
|  | 71304 | 03/09/2017 | A | F | NI | NI | Osasco | SP | 0 | 0 | 1 | 0 | *A. parkeri* | |
|  | 71515 | 16/09/2017 | A | NI | - | N | São Paulo | SP | 0 | 0 | 0 | 1 | *A. longirostre* | |
|  | 71686 | 23/09/2017 | A | NI | AP | W | São Paulo | SP | 0 | 0 | 0 | 1 | *A. longirostre* | |
|  | 71929 | 29/09/2017 | A | M | - | - | Embu das Artes | SP | 0 | 0 | 0 | 1 | *A. longirostre* | |
|  | 72191 | 08/10/2017 | A | NI | - | E | São Paulo | SP | 0 | 0 | 1 | 0 | *A. longirostre* | |
|  | 72234 | 09/10/2017 | A | F | - | - | Francisco Morato | SP | 0 | 0 | 0 | 1 | *A. longirostre* | |
|  | 72657 | 23/10/2017 | A | NI | - | S | São Paulo | SP | 0 | 0 | 1 | 0 | *A. longirostre* | |
|  | 75264 | 25/01/2018 | A | M | JSP | W | São Paulo | SP | 0 | 0 | 1 | 0 | *A. parkeri* | |
|  | 77605 | 02/06/2018 | A | F | - | - | Itapecerica da Serra | SP | 0 | 0 | 0 | 1 | *A. longirostre* | |
|  | 78008 | 04/07/2018 | A | F | - | - | Atibaia | CP | 0 | 0 | 0 | 1 | *A. longirostre* | |
|  | 78248 | 20/07/2018 | A | M | - | - | Caieiras | SP | 0 | 0 | 1 | 1 | *A. longirostre* | |
|  | 79054 | 08/09/2018 | A | F | - | - | Franco da Rocha | SP | 0 | 0 | 0 | 1 | *A. longirostre* | |
|  | 79591 | 22/09/2018 | A | M |  | - | Franco da Rocha | SP | 0 | 0 | 0 | 1 | *A. longirostre* | |
|  | 82870 | 14/12/2018 | A | F | ALSP | N | Franco da Rocha | SP | 0 | 0 | 1 | 2 | *A. longirostre* | |
|  | 82893 | 15/12/2018 | A | M | CP | E | São Paulo | SP | 0 | 3 | 0 | 0 | *A. longirostre* | |
|  |  |  |  |  |  |  |  |  | 0 | 1 | 0 | 0 | *A. parkeri* | |
|  | 83199 | 28/12/2018 | A | F | - | W | São Paulo | SP | 0 | 0 | 1 | 0 | *A. parkeri* | |
|  | 84276 | 01/03/2019 | A | F | - | S | São Paulo | SP | 0 | 0 | 3 | 0 | *A. longirostre* | |
|  | 86300 | 04/08/2019 | A | M | - | S | São Paulo | SP | 0 | 0 | 1 | 0 | *A. longirostre* | |
|  | 87373 | 30/09/2019 | A | F | - | - | Diadema | SP | 0 | 0 | 0 | 1 | *A. longirostre* | |
|  | 87398 | 01/10/2019 | A | F | - | W | São Paulo | SP | 0 | 0 | 1 | 0 | *A. longirostre* | |
|  | 87440 | 02/10/2019 | A | F | - | N | São Paulo | SP | 0 | 0 | 1 | 0 | *A. longirostre* | |
|  | 87846 | 15/10/2019 | A | F | - | - | Cotia | SP | 0 | 0 | 1 | 1 | *A. longirostre* | |
|  | 92519 | 06/06/2020 | A | M | - | N | São Paulo | SP | 0 | 0 | 1 | 1 | *A. longirostre* | |
|  | 93276 | 11/08/2020 | A | M | AP | W | São Paulo | SP | 0 | 0 | 1 | 0 | *A. longirostre* | |
|  | 94903 | 02/10/2020 | A | M | - | W | São Paulo | SP | 0 | 0 | 0 | 1 | *A. longirostre* | |
|  | 95174 | 09/10/2020 | C | F | JSP | - | Franco da Rocha | SP | 0 | 0 | 0 | 1 | *A. longirostre* | |
|  | 96395 | 22/11/2020 | A | M | - | N | São Paulo | SP | 0 | 0 | 1 | 0 | *A. longirostre* | |
|  | 99185 | 10/03/2021 | A | M | - | N | São Paulo | SP | 0 | 0 | 1 | 0 | *A. parkeri* | |
|  |  |  |  |  |  |  |  |  | 0 | 0 | 7 | 0 | *A. longirostre* | |
|  | 99521 | 04/04/2021 | A | F | - | - | Sorocaba | SO | 6 | 0 | 1 | 0 | *A. longirostre* | |
|  | 101012 | 14/07/2021 | A | F | ALSP | N | São Paulo | SP | 0 | 0 | 1 | 0 | *A. longirostre* | |
|  | 101629 | 23/08/2021 | A | M | JSP | - | Franco da Rocha | SP | 0 | 1 | 1 | 0 | *A. longirostre* | |
|  | 101671 | 25/08/2021 | A | M | JSP | - | Franco da Rocha | SP | 0 | 1 | 0 | 0 | *A. longirostre* | |
|  | 101752 | 28/08/2021 | C | F | JSP | - | Franco da Rocha | SP | 0 | 0 | 0 | 1 | *A. longirostre* | |
|  | 101799 | 31/08/2021 | A | F | - | E | São Paulo | SP | 0 | 0 | 1 | 2 | *A. longirostre* | |
|  | 102634 | 26/09/2021 | J | F | - | - | Santana de Parnaíba | SP | 0 | 1 | 1 | 0 | *A. longirostre* | |
|  |  |  |  |  |  |  |  |  | 0 | 1 | 0 | 0 | *A. ovale* | |
|  | 103612 | 20/10/2021 | A | M | - | W | São Paulo | SP | 0 | 0 | 0 | 3 | *A. longirostre* | |
|  | 105916 | 11/01/2022 | A | M | - | W | São Paulo | SP | 0 | 0 | 1 | 0 | *A. longirostre* | |
|  | 106400 | 24/01/2022 | A | F | - | S | São Paulo | SP | 0 | 0 | 1 | 0 | *A. parkeri* | |
|  | 106900 | 15/02/2022 | A | M | AP | W | São Paulo | SP | 2 | 0 | 2 | 1 | *A. longirostre* | |
|  | 108307 | 12/05/2022 | A | M | - | - | Cotia | SP | 0 | 0 | 1 | 0 | *A. parkeri* | |
|  |  |  |  |  |  |  |  |  | 0 | 0 | 1 | 0 | *A. longirostre* | |
|  | 108533 | 26/05/2022 | A | M | - | S | São Paulo | SP | 0 | 0 | 2 | 0 | *A. longirostre* | |
|  | 109584 | 03/08/2022 | NI | NI | NI | NI | São Paulo | SP | 0 | 0 | 0 | 2 | *A. longirostre* | |
|  | 109631 | 07/08/2022 | A | F | - | - | Vargem Grande Paulista | SP | 5 | 0 | 1 | 0 | *A. longirostre* | |
|  | 110128 | 30/08/2022 | A | F | - | W | São Paulo | SP | 0 | 0 | 1 | 0 | *A. longirostre* | |
|  | 111757 | 13/10/2022 | A | F | VNP | S | São Paulo | SP | 0 | 0 | 1 | 0 | *A. longirostre* | |
|  |  |  |  |  |  |  |  |  | 0 | 2 | 0 | 0 | *A. parkeri* | |
|  |  |  |  |  |  |  |  |  | 0 | 2 | 0 | 0 | *A. sculptum* | |
|  | 114074 | 19/12/2022 | J | F | - | S | São Paulo | SP | 0 | 0 | 1 | 0 | *A. longirostre* | |
|  | 115754 | 20/02/2023 | A | M | - | W | São Paulo | SP | 0 | 0 | 0 | 1 | *A. longirostre* | |
|  | 116019 | 03/03/2023 | A | F | - | - | Taboão da Serra | SP | 0 | 0 | 2 | 0 | *A. longirostre* | |
|  | 116857 | 22/04/2023 | A | F | - | - | Cajamar | SP | 0 | 0 | 0 | 1 | *A. longirostre* | |
|  | 117308 | 18/05/2023 | A | F | - | - | Barueri | SP | 0 | 0 | 0 | 1 | *A. parkeri* | |
|  | 117860 | 13/07/2023 | A | F | - | - | Jundiaí | CP | 0 | 0 | 0 | 1 | *A. longirostre* | |
|  | 118112 | 01/08/2023 | A | F | - | - | Louveira | CP | 0 | 0 | 0 | 1 | *A. longirostre* | |
|  | 118113 | 01/08/2023 | A | M | - | - | Jundiaí | CP | 0 | 0 | 1 | 0 | *A. longirostre* | |
|  | 118429 | 17/08/2023 | A | M | - | - | Cotia | SP | 0 | 0 | 5 | 0 | *A. longirostre* | |
|  | 119611 | 25/09/2023 | A | NI | - | W | São Paulo | SP | 0 | 0 | 0 | 1 | *A. longirostre* | |
|  | 119765 | 28/09/2023 | A | F |  | N | São Paulo | SP | 0 | 1 | 0 | 0 | *Amblyomma dubitatum* | |
|  | 121432 | 08/11/2023 | A | F | - | N | São Paulo | SP | 0 | 0 | 1 | 1 | *A. longirostre* | |
|  | 121529 | 10/11/2023 | A | F |  | W | São Paulo | SP | 0 | 0 | 1 | 0 | *A. longirostre* | |
|  | 124382 | 07/02/2024 | A | NI | - | S | São Paulo | SP | 0 | 0 | 0 | 2 | *A. longirostre* | |
|  | 125369 | 09/03/2024 | A | F | AP | W | São Paulo | SP | 0 | 0 | 1 | 0 | *A. longirostre* | |
|  |  |  |  |  |  |  |  |  | 0 | 0 | 1 | 0 | *A. parkeri* | |
|  | 125759 | 02/04/2024 | A | F | - | - | Jundiaí | CP | 2 | 0 | 0 | 0 | *A. longirostre* | |
|  | 126353 | 01/05/2024 | A | M | - | S | São Paulo | SP | 0 | 0 | 1 | 0 | *A. longirostre* | |
|  |  |  |  |  |  |  |  |  | 0 | 0 | 1 | 0 | *A. parkeri* | |
|  | 127991 | 02/08/2024 | C | NI | - | N | São Paulo | SP | 0 | 0 | 0 | 2 | *A. longirostre* | |
|  | 128029 | 05/08/2024 | A | M | - | E | São Paulo | SP | 0 | 0 | 2 | 1 | *A. longirostre* | |
|  | 128081 | 09/08/2024 | A | F | - | W | São Paulo | SP | 0 | 0 | 1 | 0 | *A. longirostre* | |
|  | 128223 | 16/08/2024 | A | NI | - | W | São Paulo | SP | 0 | 0 | 0 | 2 | *A. longirostre* | |
|  | 128250 | 18/08/2024 | A | M | - | - | Cotia | SP | 0 | 0 | 1 | 0 | *A. longirostre* | |
|  | 129436 | 23/09/2024 | A | M | - | W | São Paulo | SP | 0 | 0 | 2 | 0 | *A. longirostre* | |
|  | 130318 | 10/10/2024 | A | NI | VRP | W | São Paulo | SP | 0 | 0 | 0 | 2 | *A. longirostre* | |
|  | 130674 | 17/10/2024 | J | M | - | - | Mairiporã | SP | 0 | 0 | 1 | 2 | *A. longirostre* | |
|  | 134239 | 08/01/2025 | As | M | - | - | Caieiras | SP | 0 | 0 | 1 | 0 | *A. longirostre* | |
|  | 136078 | 26/02/2025 | A | F | AP | W | São Paulo | SP | 20 | 0 | 0 | 0 | *A. longirostre* | |
|  | 138561 | 06/06/2025 | A | M | - | - | Embu das Artes | SP | 0 | 0 | 1 | 0 | *A. longirostre* | |
|  |  |  |  |  |  |  |  |  | 0 | 1 | 0 | 0 | *Haemophysalis juxtakochi* | |

Legends

A – Adult; ALSP - Alberto Löfgren State Park; AP – Anhanguera Park; C – cub; CP – Carmo Park; E – east; F – female; J – juvenile; JSP - Jaraguá State Park; JSP - Juquery State Park; M – male; N – north; NI – no information; S – south; TEP – Tietê Ecological Park; VNP - Varginha Natural Park; VRP - Vila dos Remédios Park; W – west.

Geopolitical regions: São Paulo (SP), Sorocaba (SO), Bauru (BA), Marília (MA), Presidente Prudente (PP), Araçatuba (AR), São José do Rio Preto (SJRP), Ribeirão Preto (RB), Araraquara (ARR), Campinas (CP) and São José dos Campos (SJC).
